# Associations of mobile internet use and depressive symptoms with cognitive performance among Chinese adolescents: a cross-sectional study

**DOI:** 10.3389/fped.2026.1883075

**Published:** 2026-07-10

**Authors:** Danyan An, Jinfei Hou, Junsong Chen, Jie Chen, Suling Wu, Meiling Sheng

**Affiliations:** 1 Department of Respiratory Medicine, Hangzhou Children’s Hospital, Hangzhou, China; 2 Department of Rheumatology and Immunology, Hangzhou Children’s Hospital, Hangzhou, China

**Keywords:** adolescents, cognitive ability, depressive symptoms, internet use, mathematics, vocabulary

## Abstract

**Background:**

Chinese adolescents are spending more time online than ever, raising concerns about impacts on their cognitive development and mental health. This study aims to examine the association between time spent online, cognitive performance, and depressive symptoms in Chinese adolescents.

**Methods:**

We analyzed data from the 2018 wave of the China Family Panel Studies for 3,226 adolescents (aged 10–19 years). Linear regression model was used to explore the associations of daily mobile internet use, depressive symptoms, with cognitive performance (vocabulary and mathematics), with linear and non-linear mobile internet use as exposures. To explore the combined effect of mobile internet use and depressive symptoms, an interaction term between them was further added to the model. Subgroup and sensitivity analyses were conducted to check the robustness of the results.

**Results:**

The study found that approximately 14.5% of Chinese adolescents had depressive symptoms. Increased mobile internet use was associated with better vocabulary performance (*β* = 0.31; 95% CI = 0.1, 0.52), while no significant association was found with mathematics performance. Adolescents with depressive symptoms had significantly lower vocabulary (*β* = −1.49; 95% CI = −2.03, −0.95) and mathematics performance (*β* = –1.16; 95% CI = –1.58, −0.74). Notably, a significant interaction between mobile internet use and depressive symptoms was observed for vocabulary performance (*β* = 0.41; 95% CI = 0.17, 0.65).

**Conclusion:**

In this cross-sectional study of Chinese adolescents, depressive symptoms were consistently associated with both vocabulary and mathematics assessments. Mobile internet use showed a positive, modestly non-linear association with vocabulary performance. The significant interaction between depressive symptoms and mobile internet use for vocabulary performance suggests possible heterogeneity by depressive-symptom status, but this exploratory finding should be interpreted cautiously.

## Introduction

Adolescents today are more connected to the internet than ever before. Globally, nearly 80% of young people are now online, a proportion higher than in any other age group (1). In many regions, internet access and smartphone ownership have become nearly universal in the teen population, with a large fraction of adolescents reporting daily or near-constant internet use (2). In China, this digitalization has been particularly rapid among young people. National reports in China show that Internet penetration among minors increased from 93.7% in 2018 to 97.2% in 2022 (3). Adolescents integrate the online world into virtually every aspect of daily life, spending on average dozens of hours per week on internet activities (4). This ubiquity of digital media has prompted growing concern and research interest in how extensive online exposure may be affecting youths' mental health and cognitive development.

Prior reviews have suggested that the association between adolescent internet use and cognitive or academic outcomes is complex rather than uniformly harmful or beneficial (5–8). On one hand, high levels of non-educational internet use might interfere with study time, attention, and learning opportunities. For example, frequent social media or gaming can distract from homework and reduce time spent on academic activities, potentially impairing school performance (9). On the other hand, the internet also provides valuable educational resources, learning tools, and cognitive stimulation that could benefit youth. Ready access to online information and digital learning platforms may enhance knowledge and skills, and certain cognitively engaging activities (such as strategy games or creative content creation) might even strengthen problem-solving abilities. Given these countervailing possibilities, empirical studies have often found mixed or minimal effects of general internet use on adolescents’ cognitive outcomes. Several investigations in Western populations observed no significant association between time spent online and students' test scores or cognitive skills (10, 11).

Depressive symptoms represent another important context for interpreting these associations. Extensive evidence shows that adolescents with depressive symptoms frequently experience impairments in attention, memory, executive functioning, and academic achievement (12, 13). Depressive symptoms during adolescence has been associated with slower cognitive processing speed, difficulties in concentration, and reduced problem-solving skills, which can persist even after mood symptoms have subsided (14). Given the high prevalence of depressive symptoms among adolescents and its robust associations with cognitive deficits, it is critical to consider depressive symptoms when evaluating the relationship between internet use and cognitive outcomes. Failing to account for depressive symptoms could obscure important mechanisms; therefore, depressive symptoms represent a key confounding — and potentially interacting—factor that warrants explicit consideration in research on internet use and cognitive performance.

Despite growing interest in digital media, mental health, and cognition, several gaps remain. These discrepancies are likely attributable to methodological differences, including reliance on cross-sectional designs and failure to account for unmeasured confounding factors such as home environment or baseline mental health. Notably, few studies have examined how adolescents' mental health status—particularly depressive symptoms—might interact with internet use to influence cognitive performance. Most prior research has investigated the effects of digital media and adolescent depressive symptoms separately (15–17), overlooking the possibility that the cognitive consequences of internet use may vary depending on an individual's psychological context. Moreover, existing literature is heavily weighted toward Western populations, with limited evidence on Chinese adolescents, who experience distinctive educational pressures, rapid digitalization, and substantial urban-rural differences in educational and digital resources.

The present study aims to investigate the associations of mobile internet use and depressive symptoms with vocabulary and mathematics performance among Chinese adolescents using data from the 2018 wave of a nationally representative longitudinal study. We hypothesized that depressive symptoms would be inversely associated with vocabulary and mathematics performance, and that mobile internet use would show domain-specific and potentially non-linear associations with cognitive performance. We draw on data from the China Family Panel Studies (CFPS), focusing on 3,226 students aged 10–19 years. The interaction between mobile internet use and depressive symptoms was examined as an exploratory analysis.

## Method

### Study population

The research data were obtained from the CFPS, which were funded by the Peking University and carried out by the Institute of Social Science Survey (ISSS) of Peking University (18). The CFPS is a nationally representative, the annual longitudinal project reviewed and approved by the ISSS of Peking University. The CFPS surveyed approximately 15,000 households nationwide, using a multistage probability proportional-to-size sampling method, and interviewed all family members in each sampled household (19). The survey questionnaire was designed to gather individual, family, and community-level information on demographic and socioeconomic variables and information on the respondents' health conditions (20). The included data covered 37,147 Chinese individuals residing in 621 villages/communities from 25 of China's 30 provinces.

As aforementioned, the 2018 wave of the CFPS surveyed about 15,000 households and collected nearly 44,000 copies of questionnaires, of which 22% were collected by phone interviews, and the rest were collected by face-to-face interviews. The CFPS uses standardized questionnaires, computer-assisted interviewing procedures, trained interviewers, and built-in range and logic checks to improve consistency in data collection. These procedures may reduce interviewer-related variation and encourage standardized reporting; however, recall bias and social desirability bias could not be fully eliminated. According to the questionnaire of CFPS and Chinese adolescent research, we selected the participants aged 10–19 years from the respondents who completed the full 8-question version of the Center for Epidemiologic Studies Depression (CES-D) questionnaire. In the 2018 CFPS wave, information on daily mobile internet use, depressive symptoms, and cognitive performance was collected during the same survey visit, so all key variables in our analyses were measured contemporaneously in a cross-sectional design. After eliminating independent variables containing missing values, our final analytical sample consisted of 3,226 respondents in total.

The project was retrieved from the CFPS website for public access to secondary data (https://www.isss.pku.edu.cn/cfps/index.htm), which excludes all identifiable information about individual participants. All participants were asked to provide written informed consent before completing the survey.

### Mobile internet use

Participants responded to one item regarding how many hours they spend in a week using mobile internet (e.g., Smartphone, Ipad). This survey questionnaire asked “In general, how much time do you spend online on your mobile device each week?” We calculated the mean daily mobile internet use to explore the association of internet use with cognition, which facilitates the interpretation of results.

### Assessment of depressive symptoms

The CFPS used CES-D 8 (including two positive and six negative questions) (21), which contained four subscales: somatic symptoms, interpersonal relations, depressed affect, and positive affect. The respondents were asked to answer their specified emotions or behaviors in the past week: (1) I felt depressed; (2) I felt that everything I did was an effort; (3) My sleep was restless; (4) I was happy; (5) I felt lonely; (6) I enjoyed life; (7) I felt sad; (8) I could not get “going.” with the options varying from 0 to 3 (0 = never; 1 = sometimes, 1–2 days; 2 = often, 3–4 days; 3 = most of the time, 5–7 days) (22). The negative emotions were assigned 0, 1, 2, and 3, and the two positive emotions were assigned 3, 2, 1, and 0. All the scores were aggregated on a scale of 0 to 24. In this study, depressive symptoms is a persistent phenomenon, and a higher score indicates a higher level of depressive symptoms, the score of 9 was set to be the cutoff point for clinically significant depressive symptoms (22). At present, the CES-D has been deemed a practical/reliable depressive symptoms screening tool for the Chinese population. In addition, CES-D and CES-D 8 have been used in several studies in adolescents and have shown good reliability and validity (23). According to the CES-D criteria, it expressly stated the depressive symptoms were diagnosed as one week after a depressive event, rather than major depressive disorder (24). The Cronbach's alpha was 0.712 in this study, indicating good reliability of this scale.

### Assessment of cognitive performance

The cognitive performance data come from the cognitive module in the 2018 CFPS database, which includes 24 standardized mathematics questions and 34 word-recognition questions (25). These questions are all taken from standard textbooks and are arranged in ascending order of difficulty. The ability to answer the initial question depends on the level of education of the respondent, and the test is terminated upon three wrong responses in sequence. All of these questions are sorted in ascending order of difficulty, and the final score is the rank of the hardest question that the respondent answers correctly. Respondents did not know the rules before taking the test, so they would not fail on purpose. In this article, the vocabulary performance was used to measure school-related skills including word knowledge and verbal concept formation, and the mathematics performance was used to measure the ability to obtain, process, and retain mathematical information. The score ranged from 0 to 24 for the mathematics performance, 0 to 34 for the vocabulary performance.

### Assessment of covariates

In this study, potential covariates included age, gender, residence (rural vs. urban), education level, self-rated health condition, academic performance, smoke, and exercise time. Age (years) and sex (male/female) were self-reported. Residence was classified as rural vs. urban according to the CFPS household registration information. Education level was categorized into four groups based on the highest level of schooling attained: illiterate/semi-literate, primary school, middle school, and high school or above.

Self-rated health was assessed with the question “How do you rate your current health?” and responses were grouped into five categories: very healthy, healthy, somewhat healthy, fair, and unhealthy. Academic performance was measured by the item “How satisfied are you with your academic performance?” with five response options (very dissatisfied, dissatisfied, fair, satisfied, very satisfied). Smoking status was assessed with the question “Have you smoked in the past month?” and coded as yes vs. no. Exercise time was measured by asking “How many hours did you spend exercising in the past week?” and treated as a continuous variable (hours per week).

### Statistical analysis

First, descriptive analyses were utilized to describe the sample characteristics, and the data are presented as numbers (%) and mean (standard deviation, SD). The *t*-test or one-way analysis of variance (ANOVA) was performed to compare scores of vocabulary performance and mathematic performance across different groups. Second, linear and non-linear (a quadratic term) daily mobile internet use were included into the regression model to explore the associations of daily mobile internet use and depressive symptoms with cognition, with vocabulary and mathematics performance as the dependent variables and adjusting for age, sex, residence (rural/urban), education level, self-rated health, academic performance, smoking, and exercise time. Then we used these fully adjusted models to obtain the adjusted predicted values of mathematics and vocabulary performance across the observed range of daily internet use, holding all covariates at their means (for continuous variables) or reference categories (for categorical variables). The predicted values and their 95% confidence intervals were then plotted against daily mobile internet use. Third, we added interaction terms of mobile internet use and depressive symptoms to explore the potential combined effects.

Subgroup and sensitivity analyses were conducted to examine the robustness of the associations. First, to further probe potential threshold effects, daily mobile internet use was categorized into quartiles and entered as a categorical predictor in the models, with the lowest quartile serving as the reference group. Polynomial contrast tests were then performed to assess the presence and shape of linear, quadratic, and higher-order trends across quartiles. We also conducted subgroup analysis by dividing the participants by gender and age (10–15 years vs. 16–19 years) in order to investigate the correlation between mobile internet use, depressive symptoms and cognitive performance in various subgroups. In addition to the binary CES-D 8 cutoff (≥9), we conducted a sensitivity analysis treating the CES-D 8 score as a continuous variable to account for the dimensional nature of depressive symptoms.

We considered a two-sided *P*-value <0.05 as statistically significant. All analyses were conducted using Stata, version 18.0 (StataCorp LLC, College Station, Texas, USA).

## Results

### Basic characteristics of participants' mobile internet use

The 2018 CFPS included 37,354 participants. We excluded 33,137 because of age ineligibility, 991 because of missing cognitive-test data, missing mobile internet use, and missing CES-D 8 data, leaving 3,226 adolescents for analysis (Supplementary Figure S1). The mean age of the total adolescents was 14.1 (SD: 2.8) years old. A total of 466 (14.45%) adolescents had depressive symptoms. In cognitive performance, the mean vocabulary and mathematics performance were 24.9 (SD: 6.6) and 12.9 (SD: 5.4). The mean daily mobile internet use was 1.24 (SD: 1.9) hours. Statistically significant differences were observed in vocabulary and mathematics performance based on depressive symptoms status, sex, age, rural/urban residence, education level, self-rated health, and exercise time (all *p* < 0.05). Adolescents who were female, urban residents, older, had higher education levels, reported better self-rated health, and exercised more tended to achieve higher academic scores. In contrast, those reporting depressive symptoms, rural residence, lower education attainment, and poorer health status scored lower in both vocabulary and mathematics. Additionally, students who smoked had significantly higher vocabulary scores (*p* = 0.018), though this association was not seen for mathematics performance (Table 1).

**Table 1:** Baseline characteristics among 3,226 adolescents.

	*N* (%)	Vocabulary performance		Mathematics performance	
		Mean (SD)	*P*	Mean (SD)	*P*
Total	3,226 (100%)	24.89 (6.58)		12.86 (5.37)	
Mobile internet use: hours, Mean (SD)	1.24 ± 1.89	23.41 ± 6.53	*P* < 0.001	11.37 ± 4.24	*P* < 0.001
Depressive symptoms			*P* < 0.001		*P* < 0.001
No	2,760 (85.55%)	25.06 (6.28)		13.00 (5.26)	
Yes	466 (14.45%)	23.88 (8.05)		12.08 (5.95)	
Sex			*P* < 0.001		0.012
Female	1,562 (48.42%)	25.43 (6.37)		13.11 (5.48)	
Male	1,664 (51.58%)	24.38 (6.73)		12.64 (5.26)	
Age: year, Mean (SD)	14.06 ± 2.80	24.89 ± 6.58	*P* < 0.001	12.86 ± 5.37	*P* < 0.001
Residence			*P* < 0.001		*P* < 0.001
Rural	2,561 (79.39%)	24.38 (6.81)		12.40 (5.32)	
Urban	665 (20.61%)	26.83 (5.17)		14.65 (5.20)	
Education			*P* < 0.001		*P* < 0.001
Llliterate/Semi-literate	973 (30.16%)	19.74 (6.93)		8.60 (3.13)	
Primary school	1,141 (35.37%)	25.61 (5.32)		12.83 (3.87)	
Middle school	811 (25.14%)	28.15 (4.25)		16.03 (5.28)	
High school and above	301 (9.33%)	29.97 (3.34)		18.24 (5.59)	
Academic Performance			*P* < 0.001		0.077
Very dissatisfied	127 (3.94%)	22.46 (7.69)		11.01 (5.82)	
Dissatisfied	236 (7.32%)	25.18 (6.39)		13.11 (5.31)	
Fair	1,737 (53.84%)	25.36 (6.32)		13.18 (5.41)	
Satisfied	791 (24.52%)	25.35 (6.14)		13.28 (5.14)	
Very satisfied	335 (10.38%)	22.05 (7.64)		10.79 (4.96)	
Self-rated health			*P* < 0.001		0.002
Very healthy	963 (29.85%)	23.94 (6.89)		11.86 (5.13)	
Healthy	1,116 (34.59%)	25.35 (6.42)		13.52 (5.44)	
Somewhat healthy	995 (30.84%)	25.66 (5.88)		13.26 (5.23)	
Fair	103 (3.19%)	22.79 (7.64)		12.22 (5.72)	
Unhealthy	49 (1.52%)	21.65 (10.24)		10.90 (7.24)	
Smoke			0.018		0.147
No	3,156 (97.83%)	24.85 (6.61)		12.84 (5.37)	
Yes	70 (2.17%)	26.71 (4.42)		13.79 (5.19)	
Exercise time: hour, Mean (SD)	3.92 ± 7.27	23.41 ± 6.53	0.016	11.37 ± 4.24	0.001

### Relationship between Mobile internet use, depressive symptoms with cognitive function

**Figure 1:**
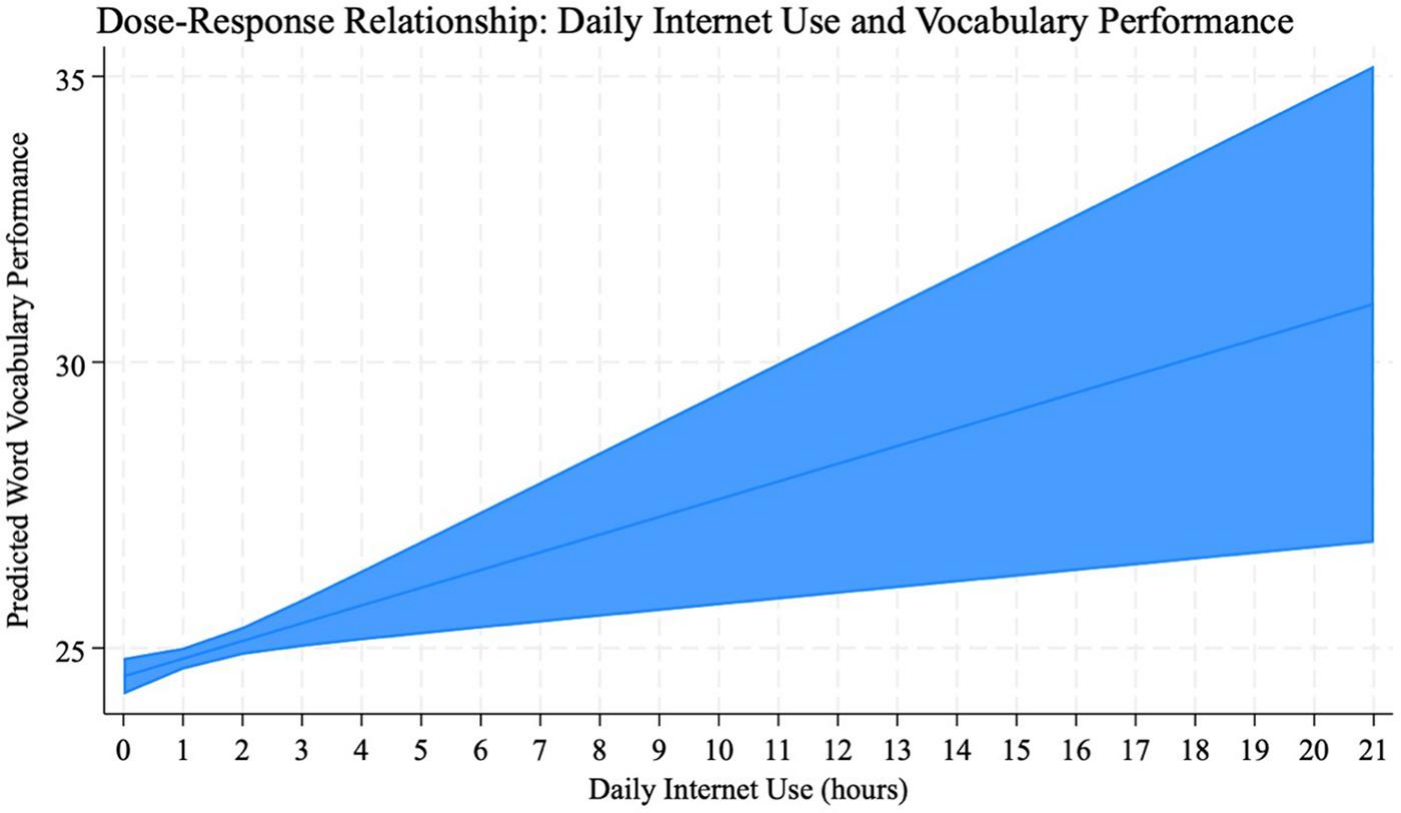
Dose response relationship between mobile internet use and vocabulary performance.

**Figure 2:**
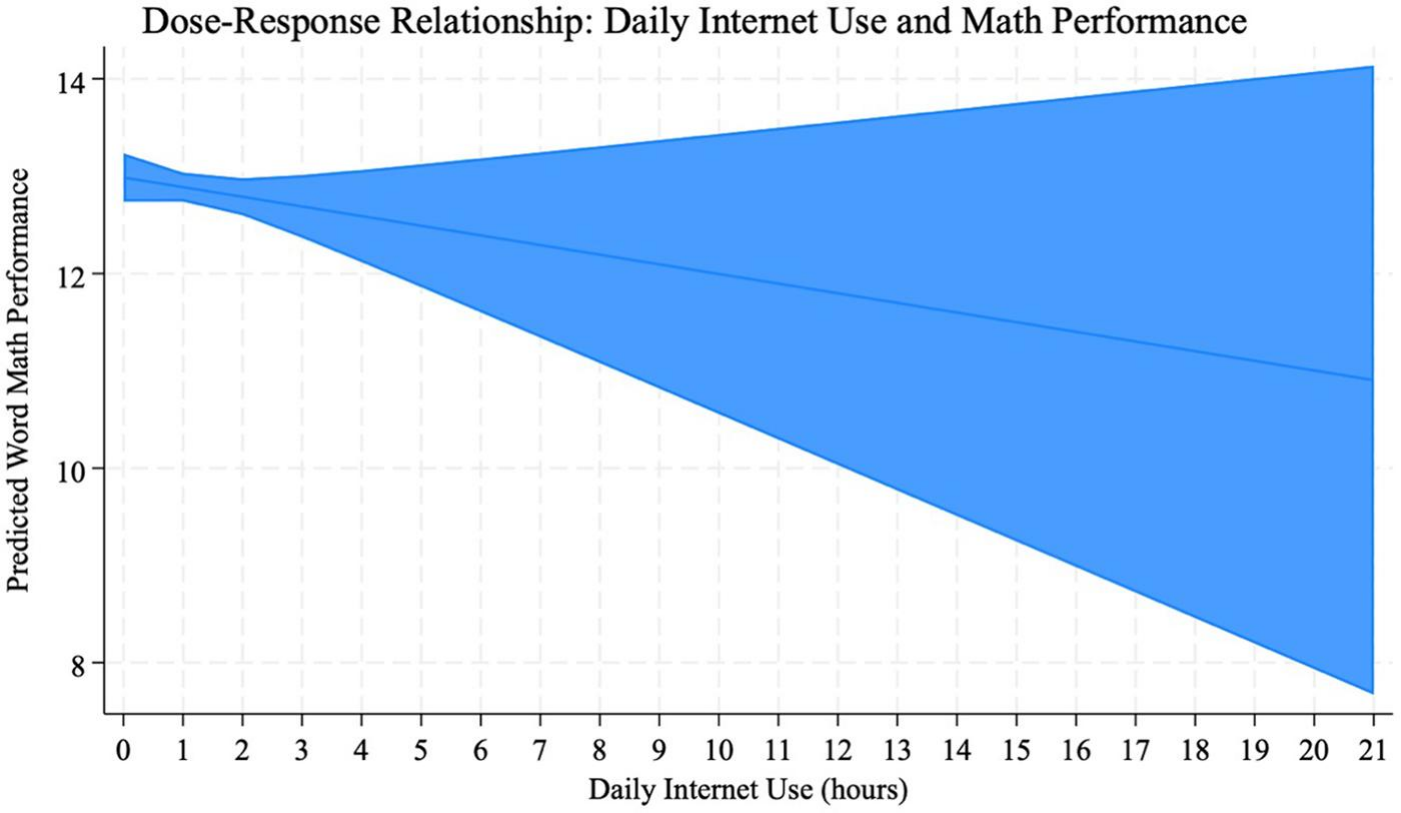
Dose response relationship between mobile internet use and mathematics performance.

After adjusting for covariables, adolescents with depressive symptoms had significantly lower vocabulary and mathematics scores (*β* = –1.49, 95% CI = –2.03 to −0.95; *β* = –1.16, 95% CI = –1.58 to −0.74, respectively). Increased mobile internet use was associated with better vocabulary performance (*β* = 0.31, 95% CI = 0.10 to 0.52) (Figure 1), but was not significantly associated with mathematics performance (*β* = −0.10, 95% CI = –0.26 to 0.06) (Figure 2). The quadratic term for daily mobile internet use was statistically significant for vocabulary performance (*β* = –0.02, 95% CI = –0.05 to −0.003), indicating that the positive association weakened as daily mobile internet use increased, but was not statistically significant for mathematics performance (*β* = –0.01, 95% CI = –0.03 to 0.004). Moreover, a significant interaction effect was observed between mobile internet use and depressive symptoms on vocabulary performance (*β* = 0.41, 95% CI = 0.17 to 0.65), suggesting that mobile internet use may have a differential impact on cognitive performance depending on depressive status (Table 2).

**Table 2:** Non-linear associations of mobile internet use, depressive symptoms with cognitive performance.

	Vocabulary performance			Mathematics performance		
	Coefficient	Lower 95% CI	Upper 95% CI	Coefficient	Lower 95% CI	Upper 95% CI
Mobile internet use	0.31**	0.1	0.52	−0.10	−0.26	0.06
Quadratic internet use time	−0.02*	−0.05	−0.003	−0.01	−0.03	0.004
Depressive symptoms (yes)	−1.49***	−2.03	−0.95	−1.16***	−1.58	−0.74
Mobile internet use # Depressive symptoms (yes)^a^	0.41**	0.17	0.65	0.15	−0.03	0.33

a Interaction effect.

* *P* < 0.05.

** *P* < 0.01.

*** *P* < 0.001.

### Subgroup and sensitivity analysis

Subgroup analyses suggested some heterogeneity by sex and age group (Table 3). By gender, the significant moderating effect of depressive symptoms on vocabulary was pronounced and significant only among males (*β* = 0.71, *p* < 0.001), with no significant effect observed for females. By age group, the 10–15-year subgroup differed from the main model, particularly for mathematics performance: both the linear term for mobile internet use (*β* = 0.36, *p* < 0.001) and the quadratic term (*β* = −0.04, *p* < 0.001) were statistically significant, whereas the corresponding associations were not statistically significant in the main model. In contrast, among adolescents aged 16–19 years, mobile internet use was negatively associated with mathematics performance, and no significant interaction with depressive symptoms was detected for either outcome.

**Table 3:** Subgroup analysis of the association between mobile internet use, depressive symptoms, and cognitive performance by gender and age group.

		Vocabulary performance			Mathematics performance		
		Coefficient	Lower 95% CI	Upper 95% CI	Coefficient	Lower 95% CI	Upper 95% CI
Female	Mobile internet use	0.34*	0.07	0.62	−0.12	−0.35	0.11
	Quadratic internet use time	−0.03	−0.05	0.00	−0.01	−0.03	0.01
	Depressive symptoms (yes)	−1.26**	−1.99	−0.53	−1.17***	−1.77	−0.58
	Mobile internet use # Depressive symptoms (yes)	0.15	−0.16	0.47	0.15	−0.11	0.40
Male	Mobile internet use	0.29	−0.06	0.64	−0.06	−0.32	0.20
	Quadratic internet use time	−0.03	−0.07	0.02	−0.02	−0.05	0.01
	Depressive symptoms (yes)	−1.74***	−2.56	−0.93	−1.13***	−1.74	−0.52
	Mobile internet use # Depressive symptoms (yes)	0.71***	0.34	1.08	0.18	−0.10	0.45
10–15	Mobile internet use	0.66***	0.34	0.98	0.36***	0.17	0.55
	Quadratic internet use time	−0.06**	−0.10	−0.03	−0.04***	−0.06	−0.02
	Depressive symptoms (yes)	−1.91***	−2.64	−1.19	−1.25***	−1.69	−0.82
	Mobile internet use # Depressive symptoms (yes)	0.49*	0.03	0.94	0.32*	0.05	0.58
16–19	Mobile internet use	−0.04	−0.31	0.22	−0.62***	−0.91	−0.32
	Quadratic internet use time	0.01	−0.02	0.03	0.02	0.00	0.05
	Depressive symptoms (yes)	−0.73	−1.51	0.04	−0.81	−1.68	0.05
	Mobile internet use # Depressive symptoms (yes)	0.24	−0.06	0.54	0.05	−0.28	0.39

* *P* < 0.05.

** *P* < 0.01.

*** *P* < 0.001.

When mobile internet use was categorized into quartiles, vocabulary scores increased from the lowest to the third quartile and then attenuated in the highest quartile, while mathematics scores were higher in the second and third quartiles but not in the highest quartile compared with the lowest quartile. Polynomial contrast tests showed significant linear (*F* = 34.29, *p* < 0.001) and quadratic (*F* = 31.28, *p* < 0.001) trends, but no cubic trend (*F* = 0.26, *p* = 0.61), suggesting a threshold or attenuation pattern in the quartile-based analysis rather than the same shape as the continuous quadratic model (Supplementary Table S1). The exploratory quartile-based associations were visualized in the supplementary materials (Supplementary Figures S2, S3). In the sensitivity analysis using continuous CES-D 8 scores, higher depressive-symptom scores were associated with lower vocabulary and mathematics performance, consistent with the main analysis using binary depressive-symptom status (Supplementary Table S2).

## Discussion

This study found that approximately 14% of Chinese adolescents in the analytic sample had elevated depressive symptoms. Greater mobile internet use was associated with higher vocabulary performance, whereas its association with mathematics performance was not statistically significant in the main model. Adolescents with depressive symptoms had lower vocabulary and mathematics scores than those without elevated depressive symptoms. We also observed an exploratory interaction between mobile internet use and depressive symptoms for vocabulary performance, suggesting that the association between internet use and vocabulary performance may differ by depressive-symptom status. Given the cross-sectional design, this finding should be interpreted as an association rather than evidence of a causal relationship.

In our nationally representative sample of Chinese adolescents, 14.5% reported significant depressive symptoms. This prevalence is on the lower end of recent estimates in China, where approximately 14%–19% of youths have been found to experience moderate depressive symptoms (26, 27). Meta-analyses indicate that the burden of adolescent depression in China has grown in recent years, with pooled prevalence around 22% (28). Our estimate thus aligns with prior findings but underscores the variability across studies due to different measures and sample characteristics. Globally, adolescent depression is recognized as a common issue: around 20%–30% of adolescents worldwide experience at least mild depressive or anxiety disorders (29).

We found that increased mobile internet use was associated with better vocabulary performance, whereas no significant association was observed with mathematics performance. This subject-specific pattern is partially reflected in existing literature. For example, Jackson et al. reported that children given home internet access showed higher reading and verbal test scores, while their math scores did not improve (30). Multiple studies have noted positive links between youths' online engagement and language skills (e.g., reading comprehension and vocabulary), alongside weaker or null links with math performance (6). These findings suggest that digital exposure is more strongly associated with verbal abilities than with quantitative skills, although the mechanisms underlying these associations cannot be determined from the present cross-sectional data. One possible explanation proposed in previous literature is that greater internet engagement may coincide with reduced study time or difficulties maintaining concentration (31). Although the association of mobile internet use with mathematics performance was not statistically significant, the direction of coefficient still suggested the potential negative association between daily mobile internet use and mathematics performance. The aforementioned meta-analysis also noted no consistent association between overall screen time and academic performance across studies (6), and highlighted substantial heterogeneity in the association between screen time and language (I^2^ = 95.5%; *p* < .001) as well as composite academic scores (I^2^ = 97.5%; *p* < .001) (6).

A notable finding in our analysis was the significant interaction between mobile internet use and depressive symptoms in relation to vocabulary performance. Adolescents with depressive symptoms who spent more time on the internet showed better vocabulary performance than depressed peers with low internet use, effectively revealing that internet use buffered the cognitive deficit (in vocabulary) associated with depressive symptoms. Directly comparable studies are scarce, as most literature has examined the effects of internet use and depressive symptoms in isolation. However, this result aligns with the broader compensation hypothesis in digital media use, which suggests that youths with mental health difficulties may turn to the internet to fulfill social and cognitive needs that are unmet offline (32). Moderate engagement online may be associated with opportunities for mental stimulation and social interaction; indeed, prior work has noted that 1–2 h of daily internet use is associated with slightly better psychological functioning in adolescents (33). For a teenager experiencing depressive symptoms—which often involves low motivation, concentration problems, and withdrawal (34)—one possible interpretation is that adolescents may use online environments as an alternative venue for interaction, information seeking, or entertainment. This could help maintain their verbal skills by exposing them to language (through text messages, social media, videos, etc.) at times when they might withdraw from real-life academic or social activities. In essence, the observed interaction may be consistent with the compensation hypothesis, although the present data cannot determine whether internet use itself contributes to differences in cognitive performance among adolescents with depressive symptoms. While caution is needed in interpreting this interaction effect, daily mobile internet use in this study was assessed with a single self-reported item and did not distinguish between educational vs. entertainment activities, social media vs. information-seeking platforms, or solitary vs. interactive use. This limitation is particularly important because digital media exposure is increasingly measurable through device-based logs, application records, or screen-time monitoring systems, whereas self-reported estimates may be imprecise. Prior work has emphasized that different kinds of screen-based and mobile activities can have divergent associations with cognitive and academic outcomes (6, 35), and that global “time-use” indicators may mask both beneficial and harmful effects. In adolescents, this issue may be further complicated by shared device use, parental control settings, and restrictions on minors' online gaming or app use, which may make individual-level exposure harder to quantify accurately. Future research should use prospective designs, objective or passively collected digital use metrics, and, where feasible, intervention designs to clarify whether specific types of mobile internet exposure are associated with cognitive outcomes.

In interpreting these findings, it is important to consider the broader Chinese sociocultural context. First, Chinese adolescents are embedded in a highly competitive, exam-oriented educational system, and many spend substantial time on digital devices for school-related tasks and after-school tutoring; this pervasive academic pressure has been linked to elevated levels of stress, burnout, and depressive symptoms (36). Second, the stigma and under-recognition surrounding mental health problems in China may lead adolescents with depressive symptoms to turn to online spaces as a relatively anonymous environment for information seeking and social interaction, potentially influencing the way depressive symptoms and internet use jointly relate to cognitive outcomes (37). Third, the stronger nonlinear associations observed among adolescents aged 10–15 years may reflect developmental differences in cognitive control, social sensitivity, learning demands, and patterns of digital-media engagement during early to middle adolescence (38, 39). At this stage, mobile internet use may include both educational opportunities and displacement of study, sleep, or offline activities; these competing pathways could produce a threshold or attenuation pattern (5, 40). Fourth, existing literature clearly shows meaningful rural-urban disparities in educational and digital infrastructure, which are likely to moderate the associations between internet use, cognition, and depressive symptoms (41). Last, in many rural areas, parental migration has created a large group of left-behind children who face heightened risks of depressive symptoms, loneliness, and problematic internet use (42), which may further shape how digital engagement and cognitive outcomes are intertwined in this population.

This study has several strengths worth highlighting. First, we leveraged data from CFPS, a rigorously conducted, nationally representative survey (19). The large, population-based sample improves the generalizability of our findings to Chinese adolescents at large, covering diverse regions and socio-demographic backgrounds. Second, our analysis considered the interaction between mobile internet use and depressive symptoms, an angle that is often overlooked. By exploring this interaction, we were able to uncover the nuanced finding that internet use may show differential associations with cognitive performance according to mental health status.

In terms of broader implications, the findings carry weight for education and youth mental health policy. The independent associations of both depressive symptoms and mobile internet use with cognitive outcomes suggest that interventions should target both domains. Schools and families in China—and globally—need to be aware that moderate, well-regulated internet use can be beneficial, whereas unregulated excessive screen time has been associated with poorer academic outcomes. At the same time, the strong link between depressive symptoms and poorer cognitive performance highlights the importance of mental health support in adolescents. Regular screening for depressive symptoms in school settings and accessible counseling services could be explored as potential approaches for supporting learning among adolescents with depressive symptoms. Although specific policy measures were beyond the scope of this study, our interaction findings suggest that well-designed online activities or e-learning platforms could potentially be leveraged to support learning among adolescents with depressive symptoms by harnessing the compensatory benefits of digital technology.

### Limitations

Despite its contributions, this study has several limitations. First and foremost is the cross-sectional design of our analysis—the data capture a single time-point, which constrains our ability to infer causality or developmental trajectories. Depressive symptoms might be a cause or a consequence of high mobile internet use. Second, daily mobile internet use was measured with a single self-reported item. Recall bias and social desirability bias may have affected self-reported variables. Adolescents may underreport behaviors or symptoms perceived as undesirable, such as prolonged mobile internet use, smoking, or depressive symptoms. Such misclassification may have attenuated the observed associations if non-differential, although the direction of bias could be unpredictable if reporting differed by psychological status, family context, or academic performance. Third, unmeasured confounding factors may exist. We controlled for several demographic covariates, but factors such as parenting style, school environment, or personality could influence both internet habits and cognitive outcomes. Last, the data were collected in 2018 because the cognitive assessment was available in that CFPS wave. Adolescent digital behavior has changed rapidly since then. National reports in China show that Internet penetration among minors increased from 93.7% in 2018 to 97.2% in 2022 (3). Therefore, the findings may not fully reflect current patterns of mobile internet use, especially after the expansion of short-video platforms, online learning, and post-pandemic digital routines.

## Conclusion

This cross-sectional study showed that depressive symptoms were associated with lower vocabulary and mathematics performance among Chinese adolescents, while mobile internet use was modestly and non-linearly associated with vocabulary performance but not mathematics performance in the main analysis. The exploratory interaction between mobile internet use and depressive symptoms for vocabulary performance suggests possible heterogeneity by depressive-symptom status, but this pattern requires confirmation in longitudinal studies with more detailed measures of digital media content, context, and timing.

## Data Availability

Publicly available datasets were analyzed in this study. This data can be found here: Institute of Social Science Survey of Peking University, https://www.isss.pku.edu.cn/cfps/index.htm.
