# Supplementary material for: Associations of mobile internet use and depressive symptoms with cognitive performance among Chinese adolescents: a cross-sectional study
Source: Front Pediatr. 2026 Jul 10;14:1883075. doi: 10.3389/fped.2026.1883075 (PMC13395873; doi:10.3389/fped.2026.1883075)
Supplement: Supplementary file 1 [file Datasheet1.docx]

Supplementary Material

# Supplementary Figures and Tables

## Supplementary Figures

**Supplementary Figure 1.** Flow diagram for the participants. CES-D 8, 8-item Center for Epidemiologic Studies Depression Scale.

**
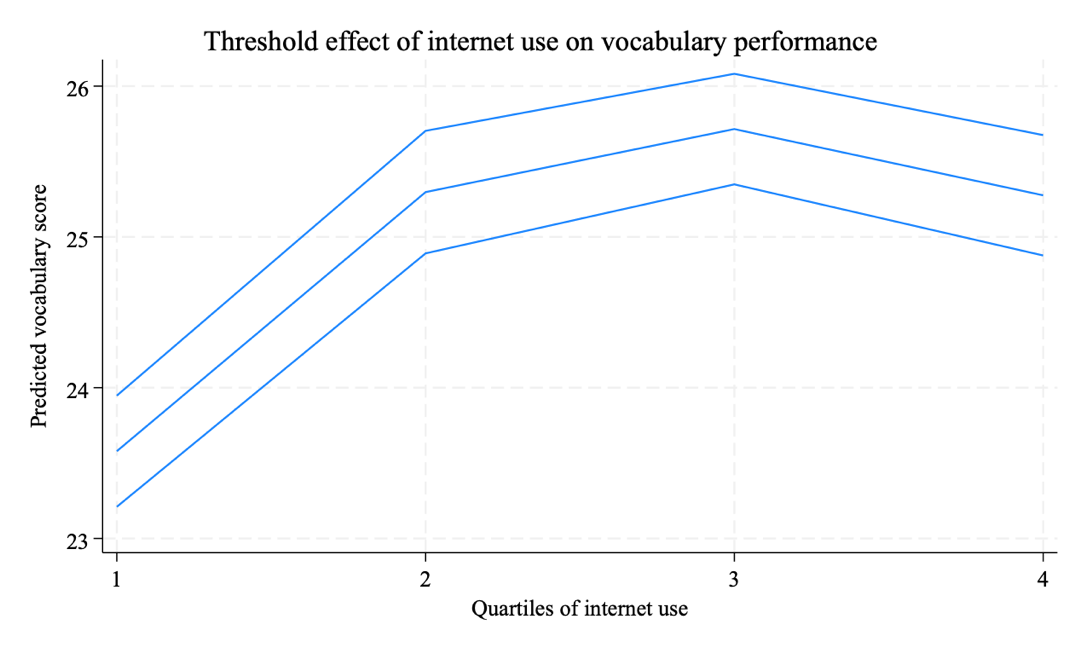
**

**Supplementary Figure 2.** Threshold effect of mobile internet use on vocabulary performance


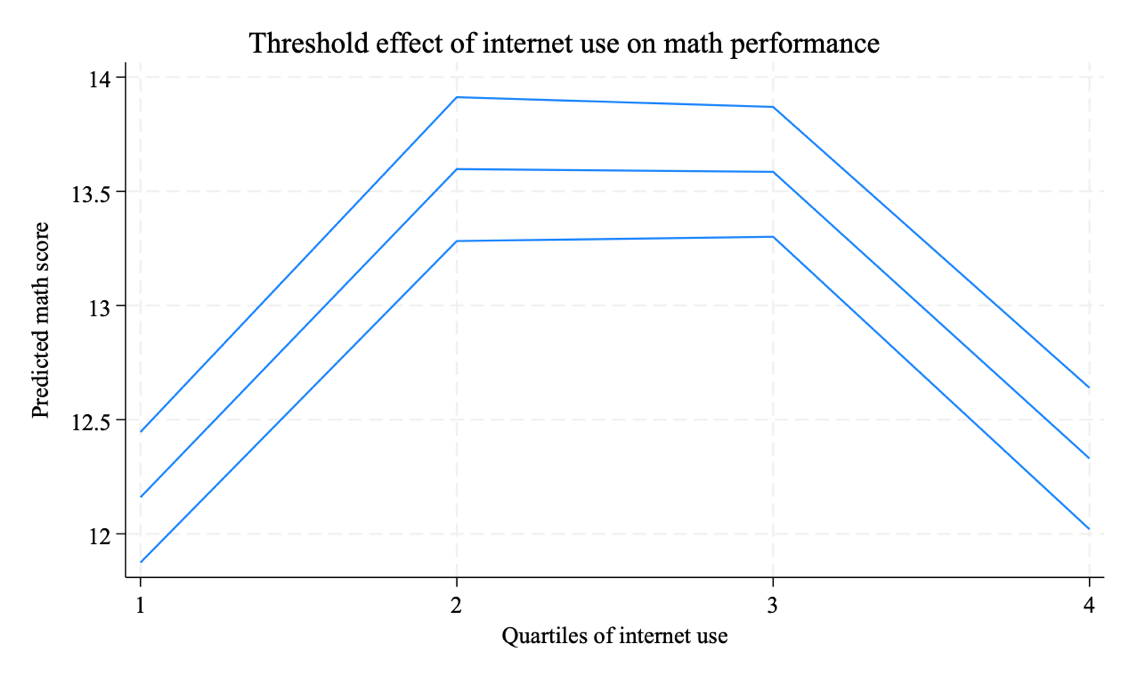


**Supplementary Figure 3.** Threshold effect of mobile internet use on mathematics performance

## Supplementary Tables

**Supplementary Table 1.** Associations of quartiles of mobile internet use, with cognition performance

|  | Vocabulary performance | | | Mathematics performance | | |
| --- | --- | --- | --- | --- | --- | --- |
|  | Coefficient | Lower 95% CI | Upper 95% CI | Coefficient | Lower 95% CI | Upper 95% CI |
| Internet use time (Ref. Quartile 1) |  |  |  |  |  |  |
| Quartile 2 | 1.72*** | 1.17 | 2.27 | 1.44*** | 1.01 | 1.86 |
| Quartile 3 | 2.14*** | 1.60 | 2.68 | 1.43*** | 1.01 | 1.84 |
| Quartile 4 | 1.7*** | 1.12 | 2.27 | 0.17 | -0.28 | 0.62 |

**Supplementary Table 2**. Sensitivity analysis using continuous CES-D 8 scores for the association between mobile internet use and cognitive performance

|  | Vocabulary scores | | | Mathematics scores | | |
| --- | --- | --- | --- | --- | --- | --- |
|  | Coefficient | Lower  95% CI | Upper  95% CI | Coefficient | Lower  95% CI | Upper  95% CI |
| Daily mobile internet use | 0.30** | 0.09 | 0.51 | -0.11 | -0.27 | 0.05 |
| Quadratic internet use time | - 0.02* | -0.04 | 0.00 | -0.01 | -0.03 | 0.01 |
| CESD-8 | -0.24*** | -0.30 | -0.17 | -0.18*** | -0.23 | -0.13 |
| Daily mobile internet use #  CESD-8 | 0.06*** | 0.03 | 0.08 | 0.01 | -0.01 | 0.03 |

* P < 0.05; ** P < 0.01; *** P < 0.001.

CES-D, Center for Epidemiologic Studies Depression.
